# Safranal Alleviated OVA-Induced Asthma Model and Inhibits Mast Cell Activation

**DOI:** 10.3389/fimmu.2021.585595

**Published:** 2021-05-20

**Authors:** Peeraphong Lertnimitphun, Wenhui Zhang, Wenwei Fu, Baican Yang, Changwu Zheng, Man Yuan, Hua Zhou, Xue Zhang, Weizhong Pei, Yue Lu, Hongxi Xu

**Affiliations:** ^1^ School of Pharmacy, Shanghai University of Traditional Chinese Medicine, Shanghai, China; ^2^ Department of Acupuncture and Moxibustion, Huachiew TCM Hospital, Bangkok, Thailand; ^3^ Shuguang Hospital, Shanghai University of Traditional Chinese Medicine, Shanghai, China; ^4^ Saffron Department and International Trade Department, Shanghai Traditional Chinese Medicine Co., Ltd., Shanghai, China

**Keywords:** *Crocus sativus*, Safranal, BMMCs, asthma, passive systemic anaphylaxis

## Abstract

**Introduction:**

Asthma is a chronic and recurring airway disease, which related to mast cell activation. Many compounds derived from Chinese herbal medicine has promising effects on stabilizing mast cells and decreasing inflammatory mediator production. Safranal, one of the active compounds from *Crocus sativus*, shows many anti-inflammatory properties. In this study, we evaluated the effect of safranal in ovalbumin (OVA)-induced asthma model. Furthermore, we investigate the effectiveness of safranal on stabilizing mast cell and inhibiting the production of inflammatory mediators in passive systemic anaphylaxis (PSA) model.

**Methods:**

OVA-induced asthma and PSA model were used to evaluate the effect of safranal *in vivo.* Lung tissues were collected for H&E, TB, IHC, and PAS staining. ELISA were used to determine level of IgE and chemokines (IL-4, IL-5, TNF-α, and IFN-γ). RNA sequencing was used to uncovers genes that safranal regulate. Bone marrow-derived mast cells (BMMCs) were used to investigate the inhibitory effect and mechanism of safranal. Cytokine production (IL-6, TNF-α, and LTC_4_) and NF-κB and MAPKs signaling pathway were assessed.

**Results:**

Safranal reduced the level of serum IgE, the number of mast cells in lung tissue were decreased and Th1/Th2 cytokine levels were normalized in OVA-induced asthma model. Furthermore, safranal inhibited BMMCs degranulation and inhibited the production of LTC_4_, IL-6, and TNF-α. Safranal inhibits NF-κB and MAPKs pathway protein phosphorylation and decreases NF-κB p65, AP-1 nuclear translocation. In the PSA model, safranal reduced the levels of histamine and LTC_4_ in serum.

**Conclusions:**

Safranal alleviates OVA-induced asthma, inhibits mast cell activation and PSA reaction. The possible mechanism occurs through the inhibition of the MAPKs and NF-κB pathways.

## Introduction

Asthma is a chronic and recurring airway disease characterized by shortness of breath and a tight feeling in the chest. In severe cases asthma attack could lead to death. Asthma is induced by the sensitization process from repeated exposure to allergens, which increased airway responsiveness. In clinical practice, first-line drugs, such as corticosteroids and bronchodilators, are used. However, more than half of the patients receiving first-line drug therapy do not respond to the treatment (1). Thus, finding new treatments for asthma is crucial. The histological integrity of asthma includes an increase in the number of goblet cells and thickening of the airway smooth muscle (2). With repeated exposure to allergen, the number of mast cells in the bronchial tissue significantly increases (3). Therefore, mast cells are well known instigators of the asthma reaction.

Mast cells are innate immune cells that are implicated in many allergic diseases, such as allergic dermatitis (4), allergic asthma (5) and systemic anaphylaxis (6). Mast cells express various cell receptors, one of which is FcϵRI. In the early phase, mast cells are activated by a specific pathogen IgE binding to an FcϵRI receptor when the mast cell interacts with specific allergens, it degranulates to release its preformed contents, which contain substances such as histamine and proteases. The activation of mast cells also initiates signaling pathways, such as the NF-κB and MAPKs pathways. These pathways lead to the transcription and production of inflammatory cytokines, i.e., IL-6, TNF-α, and LTC_4_ via the 5-LO and cPLA_2_ pathways. These inflammatory mediators increase inflammatory reactions by inducing microvascular dilation and muscular contraction, which are the cause of edema and airway narrowing during an asthma episode. If left untreated, chronic allergic inflammation can lead to remodeling in the airway (7). Therefore, it is important to control allergic inflammation in its early phase.

Mast cells also contribute to systemic anaphylaxis, which are acute and could be lethal in severe cases. Studies have shown the relationship of mast cells, which is believed to be responsible for the elevated level of histamine in anaphylaxis patients (8). While first-line treatments, such as corticosteroid and antihistamine drugs are effective in treating asthma and anaphylactic reactions, some patients do not respond to the treatments, and some patients have a high rate of recurrence. Long-term usage of these drugs produces severe side effects. However, the side effect of these drugs remained high.

Recent investigations have focused on finding new mast cell stabilizers derived from Chinese herbal medicine. Compounds from Chinese herbal medicine showed potential efficacy in stabilizing mast cells. Saffron (*Crocus sativus.*), one of the commonly used Chinese herbal medicines, has shown many promising effects for treating anxiety and cardiovascular-related diseases. However, its active ingredients and its mechanism need further examination. Safranal is one of the main active compounds derived from saffron. It has shown therapeutic effects in the treatment of Alzheimer’s disease (9) and antioxidant (10). A previous study has shown that safranal decreased airway responsiveness in the OVA-induced asthma model (11). In this study, we investigated this effect *in vivo via* the PSA model and evaluated the effect of safranal on OVA-induced asthma in mice. Furthermore, we used primary mast cells (BMMCs) to investigate the effectiveness of safranal on stabilizing mast cell degranulation and inhibiting the production of inflammatory mediators.

## Materials and Methods

### Drug and Reagents

Safranal, OVA, DNP-IgE, and DNP-HSA was purchased from Sigma Aldrich (MO, USA), purity of Safranal is ≥90.0%. RMPI-1640, fetal bovine serum (FBS), penicillin, and streptomycin were purchased from Gibco (Grand Island, NY/Carlsbad, CA, USA). Milli-Q water was supplied from a water purification system (Millipore, MA, USA). The horseradish peroxidase (HRP)-conjugated goat anti-rabbit IgG was purchased from Invitrogen (Carlsbad, CA, USA), Alum Adjuvant (Thermo Scientific, USA).

### Animal Grouping

Female BALB/c mice (18-20 g) were purchased from the Shanghai SLAC Laboratory (Shanghai, China) and housed in an SPF (specific pathogen-free) and controlled temperature (25 ± 2°C) environment with a 12-h light/dark cycle in the Shanghai University of Traditional Chinese Medicine. The animal ethics committee of Shanghai University of Traditional Chinese Medicine approved the animal experimental procedures and welfare (No. SZY201807007). Mice were provided a normal diet and drinking water and acclimated to the new environment for at least one week before the beginning of the experiment.

### Induction of OVA-Induced Asthma

Mice were randomly divided into five groups: a control group, an ovalbumin (OVA) model group, a low-dose safranal (200 mg/kg) group, a high-dose safranal (500 mg/kg) group, and a dexamethasone (DEX) group (0.5 mg/kg). Mice were sensitized by injection with OVA i.p (20 µg in PBS and alum) on day 0 and day 14 with a total volume of 200 µl. On days 15 to 21, mice were treated with vehicle, safranal or DEX p.o. q.d. On days 22, 23, and 24, mice were treated with OVA (1% in PBS) aerosolized in an airtight box for 30 min. On day 25, the mice were anesthetized with 1% pentobarbital sodium before cardiac puncture for blood collection. The lung and spleen were collected for further analysis.

### Induction of Passive Systemic Anaphylaxis

To induce PSA, mice were randomly divided into 4 groups: a normal control group, a positive control group (administered anti-DNP-IgE and vehicle), a low-concentration safranal group (administered anti-DNP-IgE, vehicle and 200 mg/kg, p.o.) and a high-concentration safranal group (administered anti-DNP-IgE, vehicle and 500 mg/kg, p.o). On the first day, we injected anti-DNP-IgE (2 µg in 100 µl PBS) i.v., and after 24 h, the mice were given safranal or vehicle for 1 h before i.v injection with DNP-HSA (2 mg in 200 µl PBS). After 5 min, the mice were anesthetized with 1% pentobarbital sodium and euthanized by cardiac puncture. Blood was collected and kept at 4°C for 4 h before centrifugation, at 3,000 rpm for 15 min. Serum was collected for further analysis.

### Culture and Activation of BMMCs

Female BALB/c mice (18–20 g) were euthanized, and the hind legs were dissected. Bone marrow was flushed with serum-free RPMI-1640, and 30% Pokeweed Mitogen-Spleen Cell Conditioned Medium (PWM-SCM) was used to culture the bone marrow cells. The medium was changed every other day. After 5 to 6 weeks, the BMMCs were mature and confirmed according to previously described procedures (12). Prior to the experiment, BMMCs were changed from culture medium to RPMI-1640 and sensitized overnight with DNP-IgE (500 ng/ml). Cells were washed with PBS and pretreated with or without safranal for 1 h before stimulation with DNP-HSA (100 ng/ml).

### Cell Viability Assay

Cell viability was determined using the MTT assay. BMMCs were seeded in 96-well plates at a concentration of 1 × 10^5^ cells/ml overnight. Cells were treated with various concentrations of safranal for 4 h before the addition of MTT (0.5 mg/ml) and incubated for 4 h. The supernatant was removed, and hydrochloride-isopropanol was added to the precipitate and mixed until fully dissolved. The OD was measured at 570 nm.

### Measurement of β-Hexosaminidase

To measure the percentage of degranulation in mast cells, BMMCs were pretreated with DNP-IgE (500 ng/ml) in HBSS medium overnight before performing the assays. Cells were washed with PBS for three times, then diluted to 1×10^6^ cells/ml, seeded in 96-well plates and treated with or without safranal for 30 min before stimulation with DNP-HSA (100 ng/ml) for 15 min. The percentage of β-hexosaminidase (β-hex) released was determined by adding substrate containing P-nitrophenyl-N-acetyl-β-d-glucosaminide (Sigma-Aldrich, MO, USA) and citric acid (pH 4.5) to the supernatant as previously described (5).

### Measurement of Inflammatory Mediators

BMMCs were seeded at 1 × 10^6^ cells/ml and incubated overnight. Safranal (10 µM or 100 µM) was added for 1 h, and the cells were stimulated with DNP-HSA for 24 h at 37°C and 5% CO_2_ in an incubator. The supernatants were collected and centrifuged at 3000 rpm for 5 min at 4°C for analysis of IFN-γ, IL-6, and TNF-α concentrations using ELISA (R&D Systems, Minneapolis, MN, USA). IgE and IL-5 levels were measured by ELISA (BD Bioscience, NJ, USA). The levels of LTC_4_ and histamine were measured using an EIA kit (Cayman Chemical, MI, USA). IL-13 and IL-4 levels were measured by an ELISA kit (Thermo Scientific, USA) according to the manufacturer’s instructions.

### Western Blot Analysis

BMMCs were homogenized, and protein levels were quantified using BCA reagent (Beyotime, Shanghai, China). Nuclear and cytoplasmic extractions were performed as instructed by the manufacturer (Beyotime, Shanghai, China). Samples were loaded and electrophoresed by 10% to 12% SDS-PAGE and transferred to nitrocellulose membranes. The membranes were blocked in 5% nonfat milk and diluted in TBS-T for 2 h, followed by incubation with primary antibodies. The following primary antibodies were used in this experiment, iNOS, COX-2, phospho-IKKα/β, IKKα/β, phospho-IκBα, IκBα, phospho-ERK½, ERK½, phospho-p38, p38, p65, phospho-c-Jun, c-Jun, c-fos, β-tubulin, lamin A/C, phospho-JNK, JNK, phospho-cPLA_2_, 5-LO, and β-actin, at a 1:1000 dilution (Cell Signaling Technology, Danvers, MA, USA). Secondary antibodies included horseradish peroxide-conjugated goat anti-rabbit antibody and anti-mouse IgG at a 1:2500 dilution.

### RNA Sequencing

BMMCs were collected after treatment with DNP-HSA with or without treatment with safranal. Total RNA was extracted using the Qiagen RNeasy Kit (Qiagen, Hombrechtikon, Switzerland), according to the manufacturer’s instructions. RNA-seq data was filtered with SOAPnuke (v1.5.2) (13). The clean reads were mapped to the reference genome using HISAT2 (v2.0.4) (14). Bowtie2 (v.2.2.5) (15) was applied to align clean reads to the reference coding gene set, and then the expression level of the gene was calculated by RSEM (v1.2.12) (16). Differential expression analysis was performed using PossionDis with false discovery rate (FDR) ≤ 0.05 and |Log2Ratio| ≥ 1. KEGG enrichment analysis of annotated differentially expressed genes was performed by Phyper based on the hypergeometric test. The sequence data were deposited in the BioSample database under the SRA accession number PRJNA639182

### Statistical Analysis

Data and statistic results are presented as the mean ± S.E.M., and all results were derived from at least three independent experiments. Statistical analyses were performed using GraphPad Prism Software 8.0 (San Diego, CA, USA). Differences between two groups were analyzed using an unpaired Student’s t test, and multiple comparisons were assessed using one-way analysis of variance (ANOVA) with Tukey’s multiple comparison test. P<0.05 was considered statistically significant.

## Results

### Safranal Alleviated OVA-Induced Asthma

**Figure 1:**
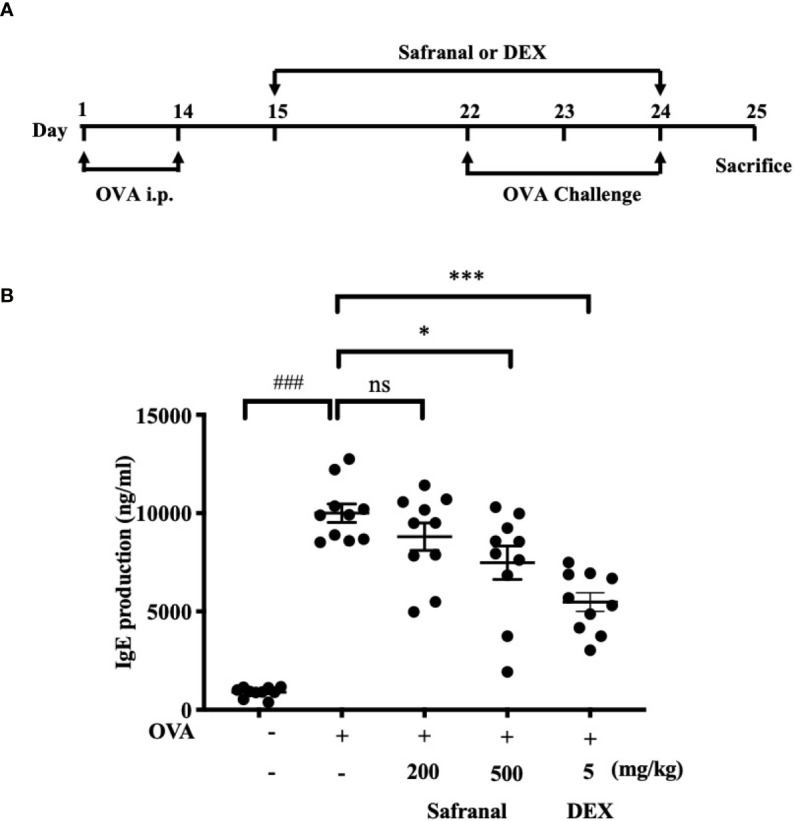
Safranal alleviates OVA-induced asthma. **(A)** OVA-induced asthma model. Mice were sensitized with OVA on days 1 and 14 before oral administration of vehicle, Safranal or Dexamethasone. On days 22, 23, and 24 Mice were challenged with OVA. Blood samples were collected by cardiac puncture and level of serum IgE was determined **(B)**. The data are presented as the means ± S.E.M. of n=10. Ns=no significance ^###^ *p* < 0.001 compared to nontreated group, *p < 0.05, ***p < 0.001 compared to OVA treated group.

**Figure 2:**
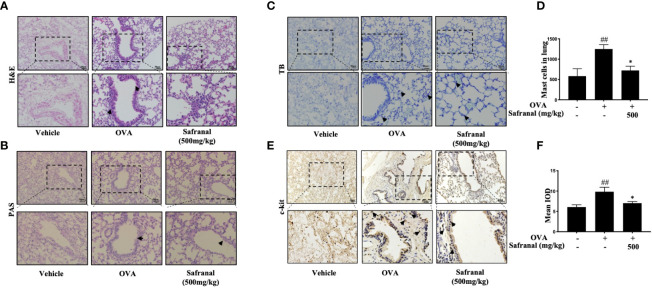
Histological of lung tissue. **(A, B)** H&E and PAS staining showed safranal suppressed infiltration of inflammatory cells, amount of mucus secretion. **(C, D)** Toluidine blue staining of lung tissue showed the number of mast cell in lung tissue. Numbers of mast cells within the lung were quantified in 200×200µm. **(E, F)** IHC staining of c-kit showed marker of the mast cells. The data are presented as the means ± S.E.M. of n=10. ^##^ *p*<0.01 compared to nontreated group. *p<0.05 compared to OVA treated group. The scale labels shown are 50 μm.

**Figure 3:**
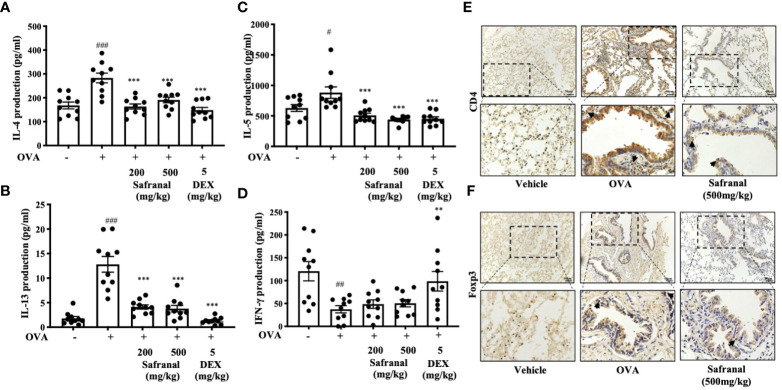
Effect of Safranal on Th1/Th2 cytokines and T cells in lung tissues. Safranal decrease the level of IL-4, IL-5, IL-13 **(A–C)** in lung tissues, while IFN-γ level increased **(D)**. Effect of safranal on T cell in lung tissues. **(E, F)** IHC staining of CD4 and Foxp3. The result showed decreased of CD4 and Foxp3 in safranal treated group. The data are presented as the means ± S.E.M. of n = 10. ^#^ *p* <0.05, ^##^ *p* <0.01, ^###^ *p* <0.001 compared to nontreated group. **p <0.01, ***p <0.001 compared to OVA treated group. The scale labels shown are 50μm.

**Figure 4:**
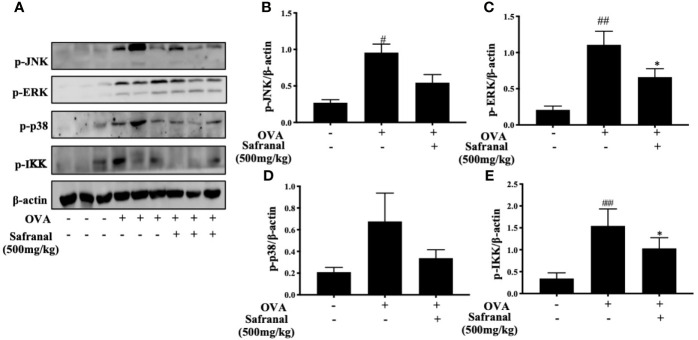
Effect of Safranal on NF-κB and MAPKs pathways in splenocyte. Safranal suppressed the activation of the NF-κB and MAPKs pathways proteins in splenocyte **(A–E)**. The data are presented as the means ± S.E.M. of n = 10. ^#^ *p* <0.05, ^##^ *p* <0.01, ^###^ *p* <0.001 compared to nontreated group. *p <0.05 compared to OVA treated group.

To investigate the effect of safranal on anti-allergic reactions, we established an OVA-induced asthma model (**Figure 1A**). After challenging with OVA, the serum IgE levels were measured. The results showed that safranal significantly reduced the IgE levels in serum (**Figure 1B**). Next, the histology of the lung tissue was evaluated *via* H&E, PAS and toluidine blue staining. We found that the infiltration of inflammatory cells and the amount of mucus secretion in the OVA-treated group were increased, while safranal suppressed the infiltration of inflammatory cells and the amount of mucus secretion (**Figures 2A, B**). The number of mast cells in the lung tissue was determined by toluidine blue staining and immunohistochemistry of c-kit. The results showed that mast cell infiltration increased significantly in the OVA-treated mice. Safranal-treated mice showed less mast cell infiltration in lung tissue than the OVA-treated mice (**Figures 2C–F**). Since Th1/Th2 cytokines are crucial in asthma, an imbalance of these cytokines can cause allergic asthma (17). We analyzed Th1/Th2 cytokine levels in lung tissue and found that the levels of IL-4, IL-5, IL-13 significantly increased, while the IFN-γ level decreased in the OVA-treated group. Safranal significantly decreased the IL-4, IL-5, and IL-13 levels (**Figures 3A–C**) and increased the IFN-γ levels in lung tissue (**Figure 3D**). This finding is consistent with the previous study (11). The production of Th1/Th2 cytokines is related to T cells. To further investigate the effect of safranal on T cells, lung tissue was stained for CD4 and Foxp3, which are markers for T cells. The results showed an increase in CD4 and Foxp3, which are CD4^+^ T cell markers in the OVA-treated group, and safranal decreased the infiltration of CD4^+^ T cells (**Figures 3E, F**). Since the spleen is an important peripheral immune organ, and it contains many types of immune cells, we further stimulated splenocyte with OVA and collected the cells for western blot analysis. We investigated whether safranal decreased the activation of the NF-κB and MAPKs pathways in splenocyte. The results showed that a high dose of safranal suppressed the activation of the NF-κB and MAPKs pathways in splenocyte (**Figures 4A–E**).

### RNA-Sequencing of Safranal and DNP-HSA–Treated BMMCs

**Figure 5:**
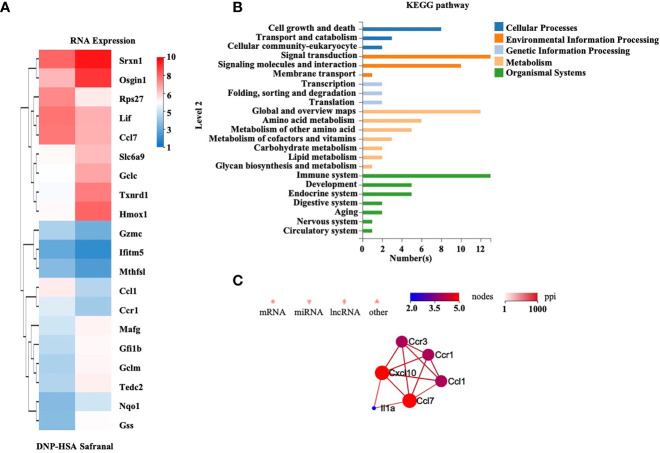
RNA-sequencing of safranal and DNP-HSA treated BMMCs. BMMCs were treated with safranal before stimulating with DNP-HSA. **(A)** Heatmap of genes expression shows effect of safranal treated BMMC on representative differentially expressed genes comparing to DNP-HSA treated. False Discovery Rate (FDR) ≤ 0.05 and |Log2Ratio| ≥ 1. **(B)** KEGG pathway database analysis found 13 differentially expressed genes that were related to the immune systems. **(C)** The protein interactions of these genes were evaluated, Ccl7 and Cxcl10 were found to be most related among the differentially expressed genes.

To alternatively uncover the genes that safranal might regulate and investigate the new potential properties of safranal, we performed RNA sequencing on antigen-activated and safranal-treated BMMCs. As the heatmap shows, the differentially expressed genes are presented in **Figure 5A** and **Supplementary 1**. We analyzed the results using the KEGG pathway database and found 13 differentially expressed genes that were related to the immune systems (**Figure 5B**). The protein interactions of these genes were evaluated, and we found that Ccl7 and Cxcl10 were highly related among the differentially expressed genes, as shown in **Figure 5C**. We also found that safranal regulated genes that are related to transient neonatal diabetes, postaxial polydactyly and allergic rhinitis (**Supplementary 2**). As result suggested that safranal regulated Cxcl10 gene, this gene is related to occurrence of asthma (18), we found that safranal decrease the level of Cxcl10 in lung tissue of OVA-treated mice (**Supplementary 3**). These chemokines may be potential target for asthma treatment and needs to be further investigated.

### Safranal Inhibited Degranulation and Decreased Inflammatory Mediator Production in BMMCs

**Figure 6:**
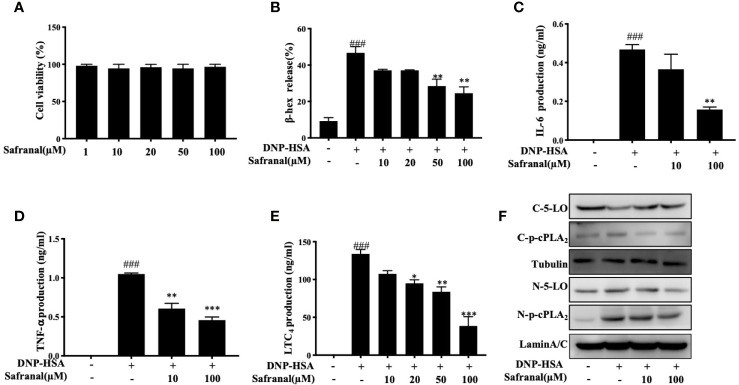
Safranal inhibited degranulation and decreased inflammatory mediator production in BMMCs. **(A)** Cytotoxicity of safranal in BMMC. Cells were treated with various concentrations of safranal, cell viability was measured using the MTT assay. **(B–E)** BMMCs were incubated with safranal and stimulation with DNP-HSA. Supernatant were collected and analyzed for β-hexosaminidase, IL-6, TNF-α, and LTC_4_ production. **(F)** Level of 5-LO and p-cPLA_2_ in cytosol and nucleus were analyzed by western blot analysis. The data shown are representative of three independent experiments. Data are the means ± S.E.M. of three independent experiments. ^###^ *p* <0.001 compared to nontreated group, *p <0.05, **p <0.01, ***p <0.001 compared to DNP-HSA.

Since safranal showed effect on reducing mast cell infiltration in OVA-induced asthma model and RNA sequencing reveals regulation of Cxcl10 and Ccl7, we proceed with the *in vitro* experiment. First, we evaluated the cytotoxicity of safranal in BMMCs by MTT assay. BMMCs were treated with various concentrations of safranal, and we found no significant cell toxicity at 100 µM (**Figure 6A**). The highest concentration of 100 µM was used in the *in vivo* experiment. Next, the degranulation of BMMCs was detected by β-hex assay. After DNP-HSA activation, mast cells released β-hex by 40% compared to that from nontreated BMMCs. We found that safranal significantly reduced the percentage of β-hex released (**Figure 6B**). While the activation of BMMCs also released cytokines, such as IL-6 and TNF-α, our results suggested that safranal significantly inhibited the production of TNF-α and IL-6 (**Figures 6C, D**). LTC_4_ is a potent cytokine released by mast cells and is related to many allergic diseases. Our results showed that safranal treatment dose-dependently decreased the production of LTC_4_ (**Figure 6E**). When activated, cPLA_2_ is phosphorylated to release arachidonic acid (AA) from the nuclear membrane, and this molecule is converted to LTC_4_ by 5-LO. We found that safranal inhibited the phosphorylation of cPLA_2_ and nuclear translocation of 5-LO (**Figure 6F**).

### Safranal Inhibited the MAPKs and NF-κB Signaling Pathways

**Figure 7:**
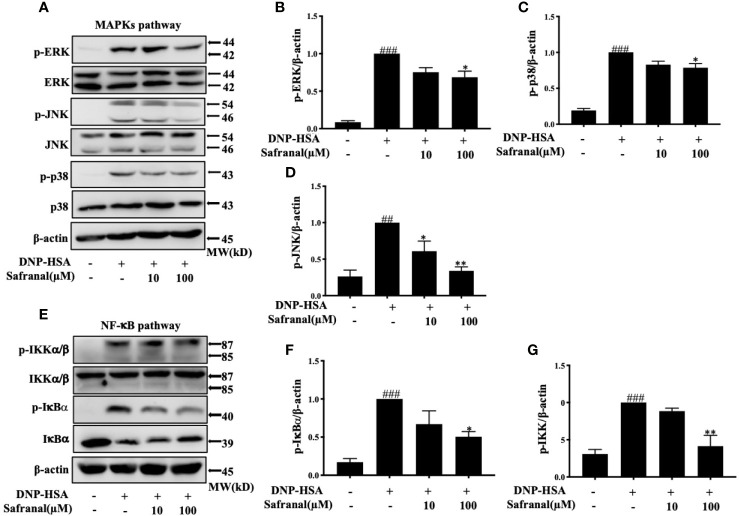
Safranal inhibited the MAPKs and NF-κB signaling pathways. Cells were pretreated with Safranal for 1 h prior to DNP-HSA treatment. The levels of phosphorylation of proteins in MAPKs pathways ERK, JNK and p38 **(A–D)** and NF-κB pathway IKK and IκBα **(E–G)** were measured by western blot and normalized to β-actin. The data shown are representative of three independent experiments and indicate the means ± S.E.M. ^##^p < 0.01, ^###^ *p* <0.001 compared to nontreated group, *p <0.05, **p <0.01compared to DNP-HSA.

**Figure 8:**
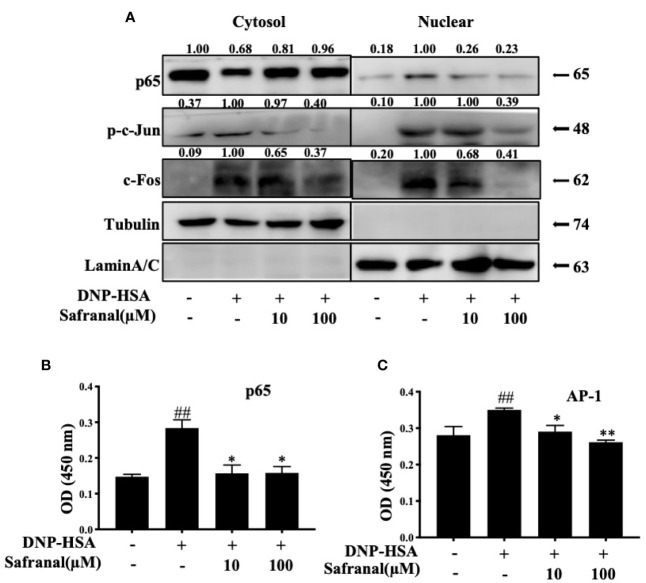
Safranal inhibited the nuclear translocation of NF-κB, AP-1. **(A)** BMMCs were collected after incubation with safranal and stimulation of DNP-HSA. Transcriptional factors p65, p-c-jun, and c-Fos in cytoplasmic or nuclear extracts were determined using western blot. **(B, C)** Nuclear transcriptional factors binding activity of p65, and AP-1 were determined by Trans AM kit, the result present in OD value. The data shown are representative of three independent experiments and indicate the means ± S.E.M. ^##^ *p* <0.01 compared to nontreated group, *p <0.05, **p <0.01 compared to DNP-HSA.

Whereas the MAPKs and NF-κB signaling pathways play a great role in inflammatory responses, our RNA-seq data showed differentially expressed genes that were related to the immune systems such as Ccl7 and Cxcl10 are related to MAPKs and NF-κB signaling pathways (19). As we previously reported, safranal inhibited the MAPKs and NF-κB signaling pathways in macrophages (20). We investigated whether safranal also inhibited these signaling pathways in BMMCs. As expected, the phosphorylation of proteins in MAPKs pathways, such as ERK, JNK, and p38, were inhibited (**Figures 7A–D**), as well as the phosphorylation of IKK and IκBα in the NF-κB pathway (**Figures 7E–G**). When both signaling pathways are activated, the transcription factors NF-κB and AP-1 are translocated into the nucleus. The transcription factors then bind to DNA and produce inflammatory mediators. We investigated whether safranal inhibited their nuclear translocation. Western blot analysis showed that the nuclear translocation of p-65 and AP-1 was inhibited (**Figure 8A**). Furthermore, we also measured the transcription factor activity *via* TransAM assay and found similar results (**Figures 8B, C**).

### Safranal Alleviated the PSA Reaction

**Figure 9:**
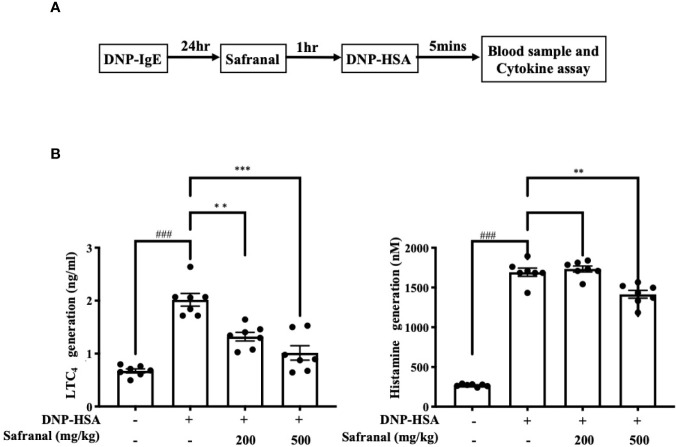
Safranal alleviated PSA reaction. **(A)** Mice were sensitized with DNP-IgE 24 h before oral administration of PBS or safranal. After 1 h mice were i.v. injected with DNP-HSA for 5 min. Blood samples were collected by cardiac puncture. **(B)** Serum was collected and analyzed for LTC_4_ and histamine production. All data shown are the means ± S.E.M. of n=7. ^###^ *p* <0.001 compared to nontreated group, **p <0.01, ***p <0.001 compared to DNP-HSA treated group.

The systemic allergic reaction is acute and deadly, and this effect is mainly due to the activation of mast cells (21). Since safranal has inhibitory effects against mast cell activation, a passive systemic anaphylaxis (PSA) model was used to investigate the anti-allergic effects of safranal *in vivo*. The levels of LTC_4_ and histamine in serum from the PSA model were analyzed. We found that the levels of LTC_4_ and histamine significantly increased in the vehicle group and decreased in the safranal-treated group (**Figures 9A, B**).

## Discussion

Research has suggested that Chinese herbal medicine has potential anti-allergic effect by inhibiting different allergic mechanisms, such as stabilizing mast cells (5), regulating eosinophils (22), balancing Treg/Th2 cells (23), and regulating the gut microbial flora (24). Saffron is commonly used in Chinese herbal medicine and has shown potential in treating diseases. Recent clinical trials suggested that saffron could be used for treating depression (25), macular degeneration (26) and asthma (27). However, its active compounds that take part in this effect are not well characterized. Active compounds derived from saffron include crocin, crocetin, picrocrocin, and safranal. Studies have reported that safranal has the potential to treat diseases such as Parkinson’s disease (28) and gastric ulcers (29). In this study, we further investigated the mechanisms involved in the anti-asthma effect of safranal.

The early asthmatic reaction is highly IgE dependent (30). Therapeutic approaches for refractory asthma include the reduction in bronchial mucosal IgE levels, and this can improve patients’ lung function (31). Since mast cells are highly associated with allergic diseases, including asthma, mast cell inhibitor showed promising results in controlling corticosteroid-dependent asthma (32). The results of this study showed that the number of mast cells increased in lung tissue and showed improvement in the integrity of lung histology. This finding suggests that safranal can reduce the migration of mast cells, thus reduce the inflammation in lung tissue. Another approach for the treatment of asthma is keeping a balance of Th2 cytokines (33). A previous report demonstrated that safranal alleviated asthma in mice by reducing iNOS levels and balancing Th2 cytokines (11). In this study, we showed safranal could reduce the production of IL-4, IL-5, and IL-13, the finding is comparable with dexamethasone. Since these cytokines are mainly produced by T cells, we further investigated the amounts of Foxp3 and CD4 in lung tissue, which are markers of T cells. These proteins were reduced in safranal-treated mice, suggested that safranal reduces the number of T cells in lung tissue. Since activation of mast cells can recruit T cells to the inflammation site (34), we suspected that safranal may decrease the number of mast cells and stabilize them to decrease the recruitment of T cells in lung tissue.

Mast cells are known to be related to allergy-related diseases, such as atopic dermatitis, asthma, systemic anaphylactic, etc. During the sensitization process, allergen specific IgE antibody is produced by B cells and binds to the FcϵRI receptor on mast cells. In the early-phase allergic reaction, specific pathogens bind to the sensitized mast cells, resulting in the degranulation and production of inflammatory mediators. Preformed substances that are deposited in mast cell granules include histamine and proteases which can cause edema and vasodilation. While the activation of pathogens also initiates the NF-κB signaling pathway, the signaling begins with phosphorylation of IKK, which leads to IκBα degradation into NF-κB p65 and p50 dimers (35). NF-κB dimers then translocate into the nucleus, the translation of dimers leads to the production of inflammatory mediators, such as IL-6 and TNF-α. Activation of specific pathogens *via* IgE-mediated reactions also leads to the phosphorylation of MAPKs, such as p38, ERK, and JNK. These MAPKs lead to nuclear translocation of AP-1, which transcribes and produces cytokines, such as IL-6 and TNF-α. Furthermore, phosphorylation of MAPKs, especially ERK (36), increases LTC_4_ biosynthesis from AA *via* the cytoplasmic cPLA_2_ and 5-LO pathway. When activated by phosphorylated ERK, cPLA_2_ frees AA from plasmid membranes. Free AA then is synthesized into LTA_4_ by 5-LO, followed by the conjugation with glutathione, which converts LTA_4_ to LTC_4_. As reported, histamine and LTC_4_ are potent mediators of anaphylactic diseases, such as asthma and systemic anaphylaxis. Studies have suggested that patients with anaphylactic shock have high levels of LTC_4_ and histamine. These mediators increase microvascular permeability, induce bronchoconstriction, etc. These are the cause of death in anaphylactic shock patients. This phenomenon is highly related to mast cell activation and degranulation. Some findings have suggested that anaphylactic reaction can be reduced by inhibiting mast cell activation (37). Several approaches have been reported to stabilized mast cells by targeting KIT (38), Fyn and Lyn (39), reducing IgE (40), etc. This study showed that safranal stabilized mast cells by decreasing degranulation and production of inflammatory mediators IL-6, TNF-α, and LTC_4_. The mechanism occurred *via* the inhibition of NF-κB and MAPKs and the inhibition of nuclear transcriptional factors. However, phosphorylated p38 and JNK protein expression in lung tissue has no statistically significant difference between model group and high dose safranal group. The results showed decreasing trend of the two proteins. Thus, further studies should be conducted to verify with greater sample size. We also found that the inhibition of MAPKs decreased LTC_4_ production by decreasing the activation and nuclear translocation of 5-LO and cPLA_2_. Since the effect of safranal on stabilizing mast cells was shown, we further investigated whether safranal could alleviate mast cell-related anaphylactic reactions. By using the PSA model, which passively stimulates mast cells *in vivo*, we found that the levels of histamine and LTC_4_ in serum were decreased.

As RNA-seq results revealed the effect of safranal on decreasing Ccl7 and Cxcl10 expression. These chemokines are related to asthma (18). Ccl7 is a chemoattractant for mast cells. By inhibiting Ccl7, the number of mast cells in lung tissue decreased (41). While 50% of asthma patients who are not responsive to corticosteroid treatments have elevated levels of Cxcl10, a study suggested that Cxcl10 could reduce steroid resistance (42). The production of Cxcl10 is related to JNK and NF-κB p65 activation (43), thus safranal could reduce Cxcl10 by the inhibition of JNK and NF-κB p65. In allergic conjunctivitis, Ccl7 promotes FcϵRI-mediated allergic reaction (44). Therefore, Ccl7 and Cxcl10 are potential targets to overcome allergic diseases. Further investigation of this mechanism should be conducted.

**Figure 10:**
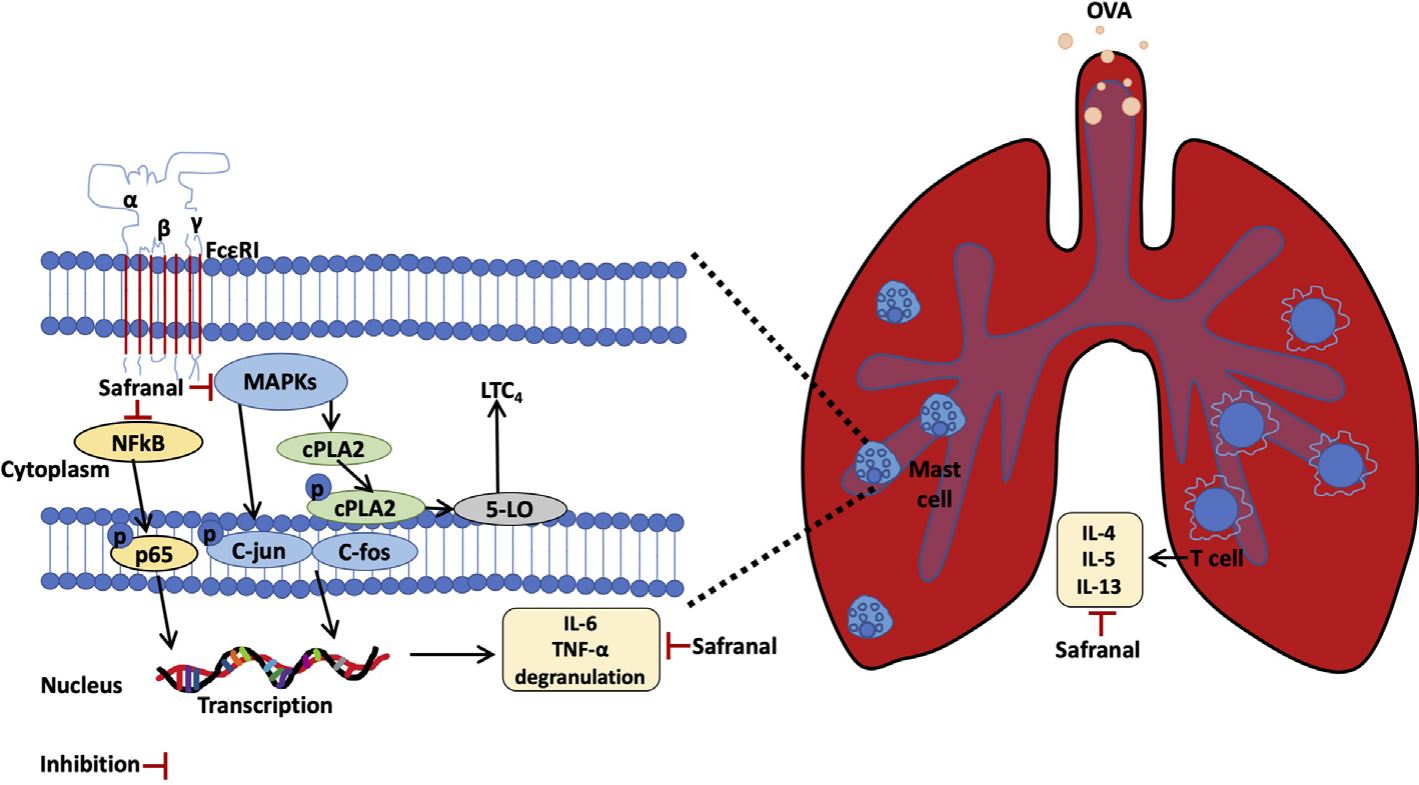
Safranal alleviated OVA-induced asthma model and inhibits mast cell activation.

Here, we report that safranal, an active compound derived from *Crocus sativus*, has the potential to treat allergic diseases such as systemic anaphylaxis and asthma. Safranal also has potential to stabilizing mast cells and inhibiting cytokine production. The possible mechanism occurs through the inhibition of the MAPKs and NF-κB pathways. As summarised in **Figure 10**.

## Data Availability Statement

The RNA-sequencing data sets presented in this study can be found in online repositories. The names of the repository/repositories and accession number(s) can be found in the article/**Supplementary Material**.

## Ethics Statement

The animal ethics committee of Shanghai University of Traditional Chinese Medicine approved the animal experimental procedures and welfare (no. SZY201807007).

## Author Contributions

HX, HZ, XZ, WP, and YL supervised the project and acquired funding. PL collected and analyzed data and investigated the results. PL and WZ collected specimens. MY, CZ, WF, and BY reviewed the data and drafts of the manuscript. All authors contributed to the article and approved the submitted version.

## Funding

This work was financially sponsored by the National Natural Science Foundation of China (NSFC) 81803545; Fok Ying-Tong Education Foundation (161039); Guangdong Province Key Area R&D Program of China (No.2020B1111110003); China-Morocco Traditional Chinese Medicine Center construction project (ZY (2018-2020)-GJHZ-1005).

## Conflict of Interest

Authors XZ and WP were employed by company Shanghai Traditional Chinese Medicine Co., Ltd.

The remaining authors declare that the research was conducted in the absence of any commercial or financial relationships that could be construed as a potential conflict of interest.

## Abbreviations

PSA, passive systemic anaphylaxis; OVA, ovalbumin; BMMCs, bone marrow-derived mast cells; LTC_4_, leukotriene C4; IL, interleukin; TNF-α, tumor necrosis factor alpha; IFN-γ, interferon gamma; NF-κB, nuclear factor-kappa B; MAPKs, a mitogen-activated protein kinase; ERK, extracellular signal-regulated kinase 1/2; JNK, c-Jun N-terminal kinase; IKK, I kappa B kinase; AP-1, activator protein 1; 5-LO, 5-lipoxygenase; cPLA_2_, cytosolic phospholipase A2; AA, arachidonic acid; HSA, human serum albumin; PWM-SCM, Pokeweed mitogen-spleen cell conditioned medium.
