# Supplementary material for: Safranal Alleviated OVA-Induced Asthma Model and Inhibits Mast Cell Activation
Source: Front Immunol. 2021 May 20;12:585595. doi: 10.3389/fimmu.2021.585595 (PMC8173045; doi:10.3389/fimmu.2021.585595)
Supplement: Supplementary file 1 [file DataSheet_1.docx]

Supplementary Material

## Supplementary Figures


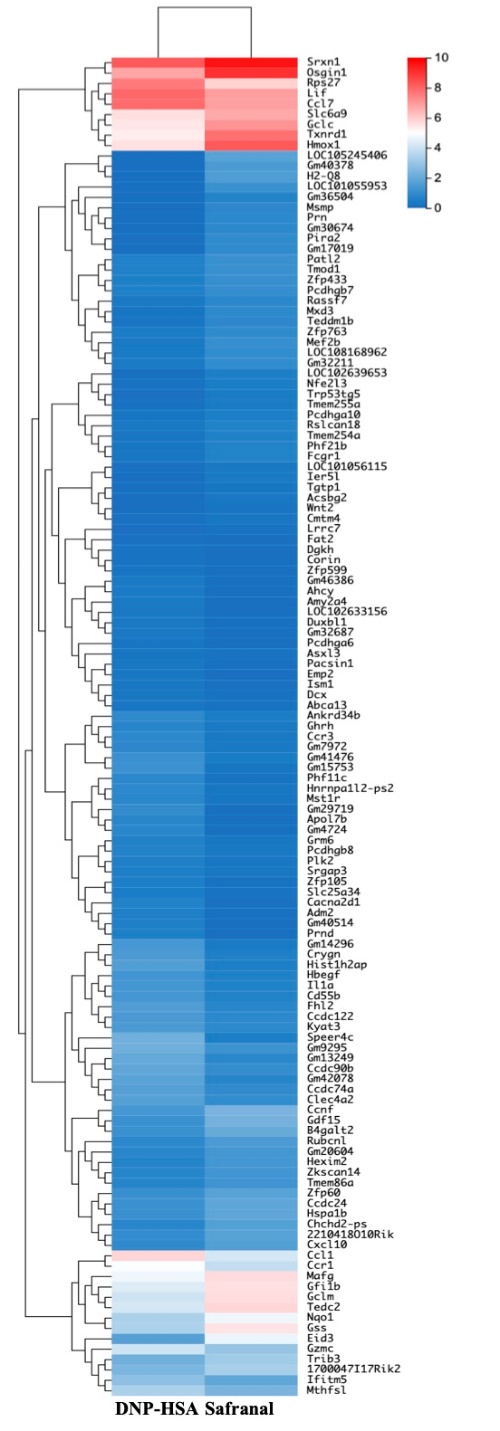


**supplementary 1 Effect of Safranal on Cxcl10 in lung tissues.** (A) Heatmap of genes expression shows effect of safranal treated BMMC on representative differentially expressed genes comparing to DNP-HSA treated. False Dicovery Rate (FDR) ≤ 0.05 and |Log2Ratio| ≥ 1.

**
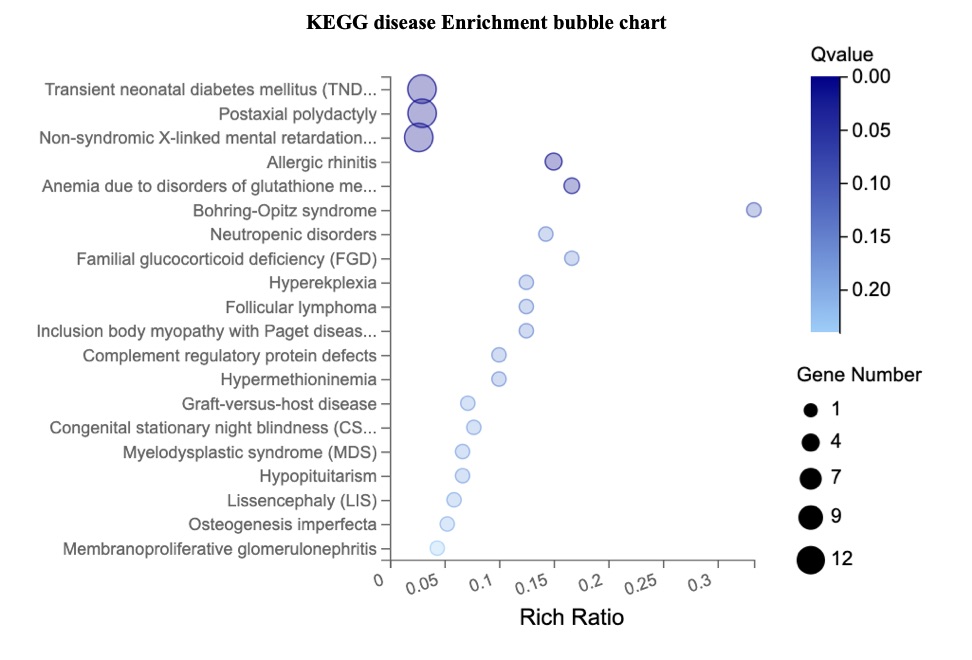
Supplementary2 KEGG diseases enrichment bubble chart.** (A) safranal regulated genes that are related to transient neonatal diabetes, postaxial polydactyly and allergic rhinitis etc.


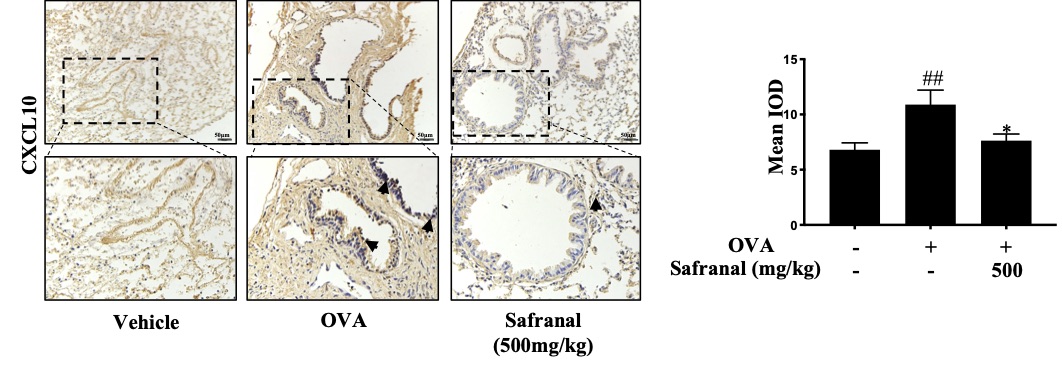


**supplementary 3 Effect of Safranal on Cxcl10 in lung tissues.** (A) IHC staining of Cxcl10. The result showed decreased of CD4 in safranal treated group. The data are presented as the means ± S.E.M. of n=10. ^##^*p*<0.01 compared to nontreated group. **p*<0.05 compared to OVA treated group. The scale labels shown are 50μm.

RNA-sequencing data can be found at: <https://www.ncbi.nlm.nih.gov/bioproject/PRJNA639182>.
