# Supplementary figures and images for: Safranal Alleviated OVA-Induced Asthma Model and Inhibits Mast Cell Activation
Source: Front Immunol. 2021 May 20;12:585595. doi: 10.3389/fimmu.2021.585595 (PMC8173045; doi:10.3389/fimmu.2021.585595)

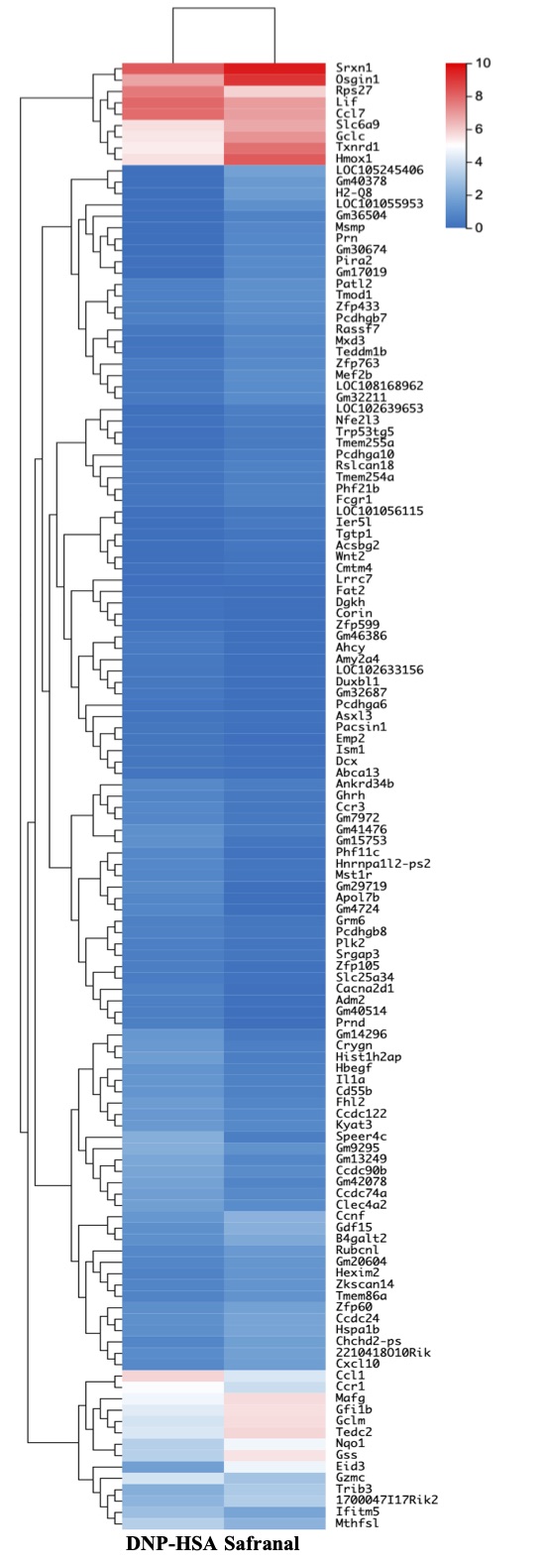

Supplement: Supplementary file 2 [file Image_1.jpg]

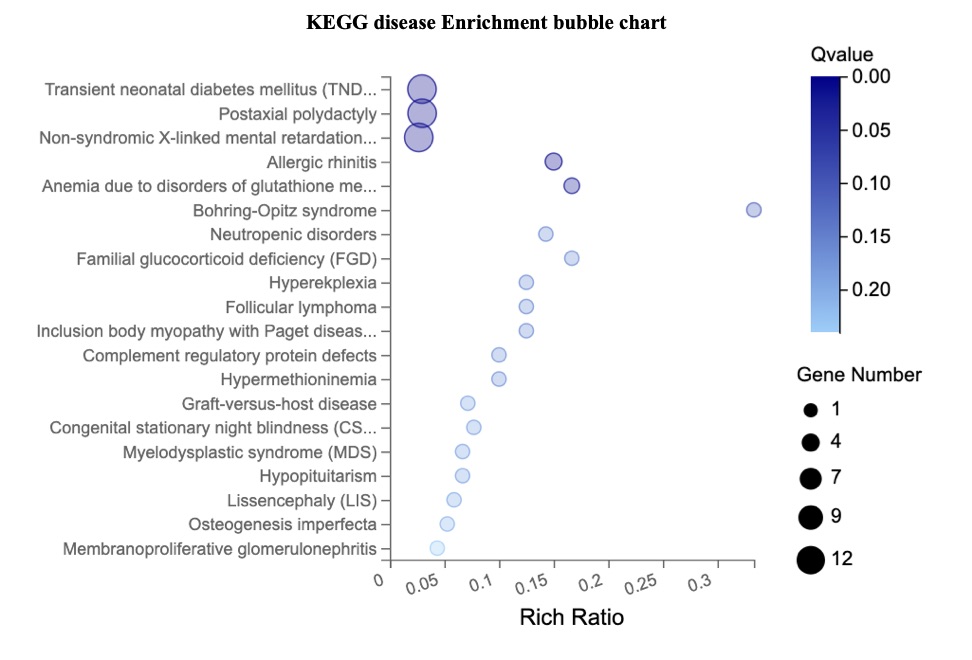

Supplement: Supplementary file 3 [file Image_2.jpg]

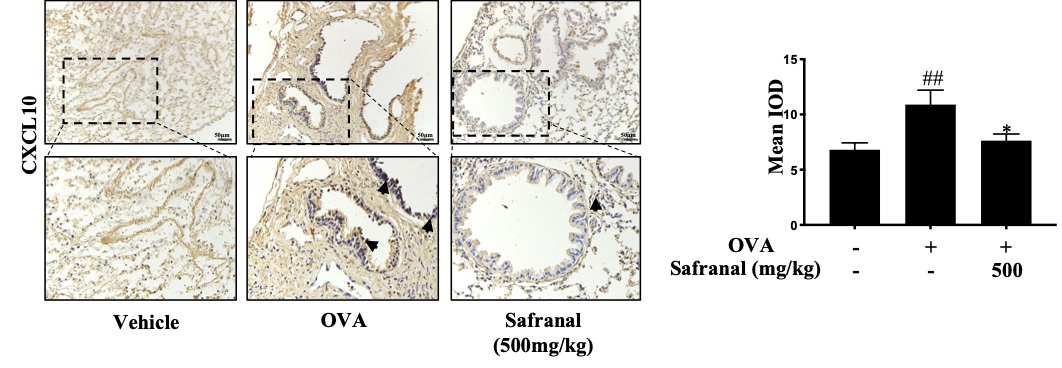

Supplement: Supplementary file 4 [file Image_3.jpg]
